# Tumor deposits counted as positive lymph nodes in TNM staging for advanced colorectal cancer: a retrospective multicenter study

**DOI:** 10.18632/oncotarget.7756

**Published:** 2016-02-26

**Authors:** Jun Li, Shengke Yang, Junjie Hu, Hao Liu, Feng Du, Jie Yin, Sai Liu, Ci Li, Shasha Xing, Jiatian Yuan, Bo Lv, Jun Fan, Shusheng Leng, Xin Zhang, Bing Wang

**Affiliations:** ^1^ General Surgery Department, Affiliated Hospital/Clinical Medical College of Chengdu University, Chengdu, People's Republic of China; ^2^ General Surgery Department, Sichuan Cancer Hospital/Institution, Chengdu, People's Republic of China; ^3^ Gastrointestinal Tumor Surgery Department, Hubei Cancer Hospital, Wuhan, People's Republic of China; ^4^ General Surgery Department, 2nd Affiliated Hospital of Jilin University, Changchun, People's Republic of China; ^5^ Internal Medicine-Oncology, Cancer Institute/Hospital, Peking Union Medical College and Chinese Academy of Medical Sciences, Beijing, People's Republic of China; ^6^ General Surgery Department, Xuzhou Central Hospital, Xuzhou, People's Republic of China; ^7^ Surgical Department of Gastrointestinal Diseases, Youan Hospital of Capital Medical University, Beijing, People's Republic of China; ^8^ Department of Pathology, Affiliated Hospital/Clinical Medical College of Chengdu University, Chengdu, People's Republic of China; ^9^ Central Laboratory, Affiliated Hospital/Clinical Medical College of Chengdu University, Chengdu, People's Republic of China

**Keywords:** colorectal cancer, tumor deposits, lymph nodes

## Abstract

We investigated the possibility of counting tumor deposits (TDs) as positive lymph nodes (pLNs) in the pN category and evaluated its prognostic value for colorectal cancer (CRC) patients. A new pN category (npN category) was calculated using the numbers of pLNs plus TDs. The npN category included 4 tiers: npN1a (1 tumor node), npN1b (2-3 tumor nodes), npN2a (4-6 tumor nodes), and npN2b (≥7 tumor nodes). We identified 4,121 locally advanced CRC patients, including 717 (11.02%) cases with TDs. Univariate and multivariate analyses were performed to evaluate the disease-free and overall survival (DFS and OS) for npN and pN categories. Multivariate analysis showed that the npN and pN categories were both independent prognostic factors for DFS (HR 1.614, 95% CI 1.541 to 1.673; HR 1.604, 95% CI 1.533 to 1.679) and OS (HR 1.633, 95% CI 1.550 to 1.720; HR 1.470, 95% CI 1.410 to 1.532). However, the npN category was superior to the pN category by Harrell's C statistic. We conclude that it is thus feasible to consider TDs as positive lymph nodes in the pN category when evaluating the prognoses of CRC patients, and the npN category is potentially superior to the TNM (7th edition) pN category for predicting DFS and OS among advanced CRC patients.

## INTRODUCTION

The TNM staging system for colorectal cancer (CRC) has been changed several times on the basis of a small expert panel consensus. The 5th edition TNM (TNM5) classification was the first to evaluate tumor deposits (TDs) and proposed the 3-mm rule in 1997 [1, 2]. The next edition (TNM6) concerned the presence of TDs in mesorectal and pericolic fat with the primary tumor, and defined TDs as positive lymph nodes (pLNs) when they had the form and smooth contour of lymph nodes (LNs) while irregular TDs with venous invasion remained in the T category [3, 4]. Recently, the presence of TDs has been reported as an important prognostic factor [5-9].

TDs were again taken into account in the American Joint Committee on Cancer (AJCC) 7th edition TNM classification (TNM7) for CRC, and a new pN1c category was proposed which states that T1 and T2 lesions that lack regional positive lymph node(s) but have tumor deposit(s) will be classified in addition as pN1c. However, it is not consistent in that in pN1c is also an option for pT3/4a tumors in the CRC staging table [10]. Despite the fact that TNM7 states that the number of TDs should be counted according to this categorization strategy, it does not point out the association of the number of TDs with stage III. There are also no clear guidelines on how to classify TDs in patients with pLNs and TDs simultaneously. This potentially impacts the accuracy of the classification and evaluation of the prognosis of CRC patients.

Recently, there has been discussion of the feasibility of TDs being counted as positive lymph nodes in the TNM staging system for CRC. Belt EJ et al. [11] declared lymph node negative CRC (stage II) with TDs should be classified and treated as stage III. Song YX et al. [5] reported that the counting of TDs as pLNs in the TNM staging system is potentially superior to the classification in the TNM7 to assess prognosis and survival for CRC patients. However, both of these studies included small numbers of patients (870 and 513 cases, respectively). In addition, in TNM7 gastric cancer staging, pathologic assessment of the regional pLNs entails their removal and histologic examination to evaluate the total number of nodes and TDs without evidence of residual LN tissue that were considered as pLN [12]. Thus, it is necessary to provide more effective data to validate the feasibility of counting the number of TDs as pLNs in the TNM classification criteria.

Here, we collected large-scale and multicenter data consisting of 4,121 stage II and III CRC patients who received initial radical surgery in order to investigate whether TDs can be counted as metastatic LNs using the AJCC TNM7 staging system for stage III CRC by calculating and comparing the 5-year disease-free survival (DFS) and overall survival (OS).

## RESULTS

A total of 4,456 patients with CRC experienced initial radical surgery. According to the exclusion criteria, 180 cases with pTis/T1 stage, 45 with simultaneous distant metastasis, and 110 with other reasons were excluded. Finally, 4,121 cases were included in this retrospective study.

### Clinicopathological characteristics of patients

In total, we identified 17.4% (717/4,121) of patients with TDs. The male: female ratio was 1.33:1 (2,352/1,769). The median age was 58.0 ± 12.1 years (range: 14-87). Clinicopathological features are listed in Table 1. In pN category (7th), the percentages of pN0-2b were 50.2%, 12.9%, 13.1%, 6.8%, 9.1% and 7.8%, respectively (*P* < 0.0001). By contrast, the percentages of npN0-2b were 50.2%, 12.8%, 15.3%, 12.0% and 9.7%, respectively (*P* < 0.0001). TDs were associated with preoperative carcinoembryonic antigen (CEA) or carbohydrate antigen 19-9 (CA19-9) levels, pT or pN category, npN category, differentiation grade, pathological category, and histological type (all *P* < 0.05). Patients with and without TDs were similar with respect to gender, age, tumor location (colon *vs*. rectum), and tumor size (diameter) (all *P* > 0.05). Additionally, the rates of patients with or without TDs who received adjuvant therapy were 17.0% and 18.2% (*P* = 0.343).

**Table 1:** Association of TDs with clinical and pathological characteristics

Variable	All Patients (*n* = 4121)		Patients with TDs (*n* = 717)		*X*^2^	*P*
	No.	%	No.	%		
Gender						
Male	2352	57.1	411	17.5	0.022	0.882
Female	1769	42.9	306	17.3		
Age, year						
≤60	2312	56.1	407	17.6	0.154	0.695
>60	1809	43.9	310	17.1		
Tumor location						
Colon	1449	35.2	278	19.2	0.51	0.475
Rectum	2671	64.8	439	16.4		
Tumor size, diameter						
≤5.0 cm	2866	69.5	508	17.7	0.68	0.410
>5.0 cm	1254	30.4	209	16.7		
Preoperative CEA levels						
<5.0 ng/ml	2479	60.2	360	14.5	39.429	<0.0001
≥5.0 ng/ml	1161	28.2	238	20.5		
Unknown	481	11.7	119	24.7		
Preoperative CA199 levels						
<29.0 u/ml	2820	68.4	465	16.5	8.565	0.014
≥29.0 u/ml	459	11.1	101	22		
Unknown	842	20.4	151	17.9		
pT category (7th)						
pT 2	128	3.1	4	3.1	66.154	<0.0001
pT 3	2851	69.2	323	11.3		
pT 4	2242	54.4	390	17.4		
pN category (7th)						
pN 0	2070	50.2	0	0	61.773	<0.0001
pN 1a	533	12.9	100	18.8		
pN 1b	539	13.1	124	23		
pN 1c	282	6.8	282	100		
pN 2a	374	9.1	80	21.4		
pN 2b	323	7.8	131	40.6		
npN category						
npN 0	2070	50.2	0	0	128.185	<0.0001
npN 1a	526	12.8	93	17.7		
npN 1b	629	15.3	214	34		
npN 2a	495	12	201	40.6		
npN 2b	401	9.7	209	52.1		
Venous invasion						
Yes	268	6.5	87	32.5	70.306	<0.0001
No	3853	93.5	630	16.4		
Lymphatic invasion						
Yes	26	0.6	12	46.2	11.528	0.001
No	4095	99.4	705	17.2		
Differentiation grade						
Well	452	11	43	9.5	90.633	<0.0001
Moderately	3213	78	523	16.3		
Poorly	456	11.1	146	32		
Pathological category						
Papillary or tubular adenocarcinoma	3856	93.6	660	17.1	8.991	0.003
Mucinous adenocarcinoma	220	5.3	38	17.3		
Signet ring cell cancer	45	1.1	19	42.2		
Histological type						
Protrude	2733	66.3	477	17.5	17.184	<0.0001
Ulcer	1151	27.9	177	15.4		
Infiltrative	237	5.8	63	26.6		
Adjuvant therapy						
Yes	2796	67.8	476	17	0.899	0.343
No	1325	32.2	241	18.2		

### TDs resulted in stage migration

A total of 1,798 TDs were detected in 717 (17.4%) patients according to the 3-mm (TNM5) and contour (TNM6) rules. The mean TD diameter was 8.5 ± 3.2 mm (range: 3-24 mm). By changing the definition of TDs to being counted as positive LNs, stage migration was likely to happen. Not surprisingly, the presence of TDs was associated with advanced npN category as compared to pN category (Table 2). TDs also more often presented with higher pT category (Table 1). In Table 2, we list stage migrations resulting from changes in the definition of TDs. Upstaging occurred in 330 of 717 patients (46.0%) with TDs that were in the pN category.

**Table 2:** pN stage migration according to TDs counted as pLNs

pN Category	npN Category				Sum
	npN1a	npN1b	npN2a	npN2b	
pN1a	433	74	18	8	533
pN1b		458	63	18	539
pN1c	93	97	85	7	282
pN2a			329	45	374
pN2b				323	323
Sum	526	629	495	401	2051

### npN as a prognostic factor for DFS and OS

During follow-up, a total of 1215 patients (29.5%) suffered failure including 351 (8.5%) with local recurrence (LR), 973 (23.6%) with distant metastasis (DM) and 109 (2.6%) with both LR and DM. The 5-year DFS and OS rates for all 4,121 patients were 69.5% and 75.2%. The clinical and pathological data including the number of LR, DM, and all failures are listed in Table 2. The 5-year DFS rates for npN0-2b were 83.6%, 72.4%, 65.6%, 45.7% and 26.0%, respectively (*P* < 0.0001). By contrast, the 5-year DFS rates for pN0-2b were 83.6%, 71.4%, 57.8%, 69.9%, 39.5%, and 25.8%, respectively (*P* < 0.0001). The 5-year OS for npN0-2b were 87.9%, 76.2%, 69.1%, 57.9% and 37.1%, respectively (*P* < 0.0001). Compared to the npN category, the 5-year OS for pN0-2b were 87.9%, 74.3%%, 64.8%, 75.2%,50.1%, and 32.9%, respectively (*P* < 0.0001).

**Figure 1:**
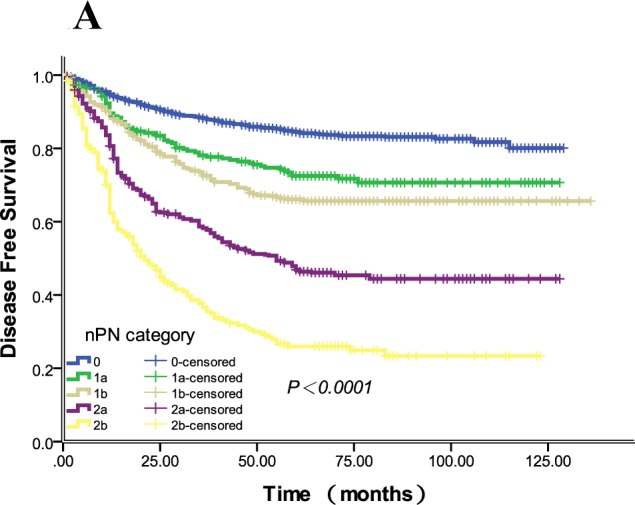

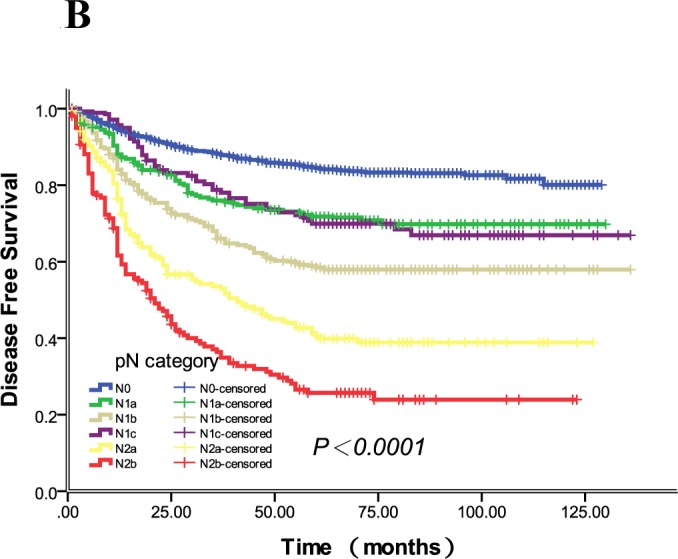

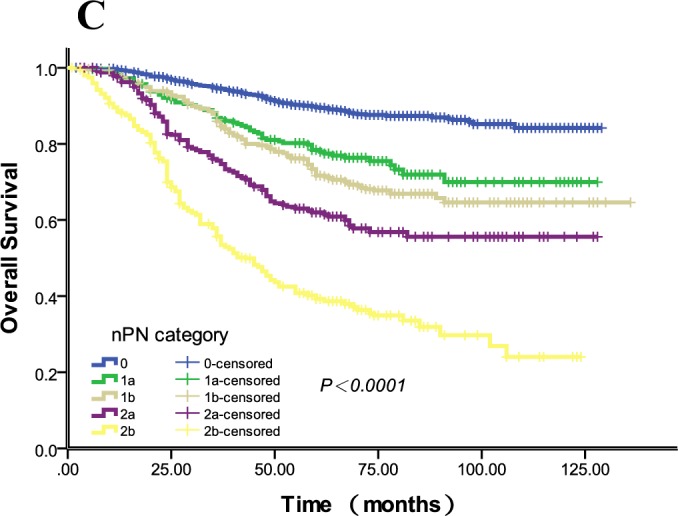

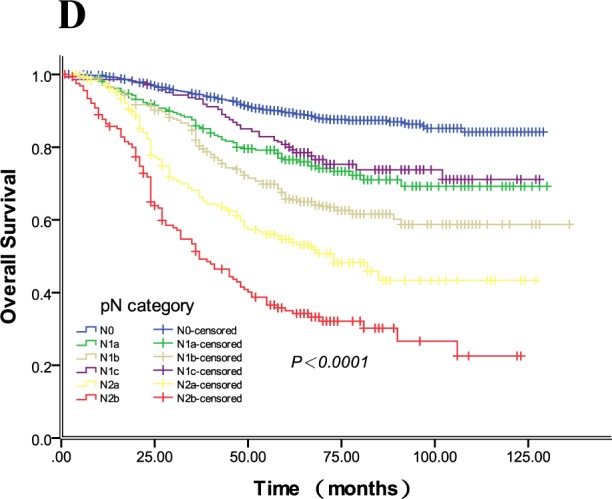
The DFS and OS curves for npN and pN categories **1A.** The 5-year DFS rates of npN0-2b were 83.6%, 72.4%, 65.6%, 45.7%, 26.0%,respectively (*P* < 0.0001), and the 5-year DFS rates of each group from npN 0 to 2b were different (all *P* < 0.05). **1B.** The 5-year DFS rates of pN0-2b were 83.6%, 71.4%, 57.8%, 69.9%, 39.5%, and 25.8%, respectively (*P* < 0.0001). pN1a and 1c had similar 5-year DFS rates (*P* = 0.862). **1C.** The 5-year OS rates of npN0-2b were 87.9%, 76.2%, 69.1%, 57.9% and 37.1%, respectively (*P* < 0.0001), and the 5-year DFS rates of each group from npN 0 to 2b were different (all *P* < 0.05). **1D.** The 5-year OS rates of pN0-2b were 87.9%, 74.3%, 64.8%, 75.2%, 50.1% and 32.9%, respectively (*P* < 0.0001), and the 5-year DFS rates of each group from pN 0 to 2b were different (all *P* < 0.05). Of note, pN1c had a similar 5-year OS rate with pN1a (*P* = 0.303).

Univariate analysis showed that the preoperative CEA or CA199 levels, pT, pN, npN, TDs, venous or lymphatic invasion, differentiation grade, pathological category and histological type were all correlated with DFS and OS (all *P* < 0.0001). Additionally, age and adjuvant chemotherapy were prognostic factors for OS but not DFS. The DFS and OS curves for both npN and pN are shown in Figure 1. Considering the fact that the npN category can be considered as an adjusted classification of the pN category, making the pN and npN categories highly correlated, multivariate models for all patients were calculated separately for each variable to avoid potential bias (Tables 4, 5). As the result, both the npN and pN categories were identified as independent prognostic factors for DFS (HR 1.614, 95% CI 1.541 to 1.673; HR 1.604, 95% CI 1.533 to 1.679) and OS (HR 1.633, 95% CI 1.550 to 1.720; HR 1.470, 95% CI 1.410 to 1.532) by multivariate analyses (all *P* < 0.0001).

**Table 3:** Influence of different clinical and pathological factors on 5-year DFS and OS

Variable	No. of All patients	Local Recurrence		Distant Metastasis		All Failure		5-Years DFS Rate	*P*	5-Year OS Rate	*P*
		No.	%	No.	%	No.	%				
	4121	351	8.5%	973	23.6%	1215	29.5%	69.5%		75.2%	
Gender											
Male	2352	219	9.3%	553	23.5%	713	30.3%	68.7%	0.081	74.9%	0.762
Female	1769	132	7.5%	420	24.7%	502	29.4%	70.6%		75.5%	
Age, year											
≤60	2312	173	7.5%	535	24.1%	652	29.2%	71.5%	0.087	78.0%	<0.0001
>60	1809	178	9.8%	438	24.2%	563	31.1%	66.9%		71.5%	
Tumor location											
Colon	1449	34	2.3%	364	25.1%	381	26.3%	73.2%	0.065	76.1%	0.132
Rectum	2671	317	11.9%	609	22.8%	834	31.2%	67.4%		74.7%	
Tumor size, diameter											
≤5.0 cm	2866	240	8.4%	670	23.4%	835	29.1%	70.1%	0.654	75.8%	0.251
>5.0 cm	1254	111	8.9%	303	24.1%	380	30.3%	68.2%		73.9%	
Preoperative CEA levels											
<5.0 ng/ml	2479	98	8.4%	463	18.7%	610	24.6%	75.0%	<0.0001	80.7%	<0.0001
≥5.0 ng/ml	1161	198	8.0%	401	34.5%	454	39.1%	58.6%		64.3%	
Unknown	481	55	11.4%	109	22.7%	151	31.4%	67.0%		72.0%	
Preoperative CA199 levels											
<29.0 u/ml	2820	32	7.0%	490	20.8%	601	26.5%	73.1%	<0.0001	81.3%	<0.0001
≥29.0 u/ml	459	169	7.2%	179	39.0%	195	42.5%	54.9%		54.5%	
Unknown	842	55	6.5%	109	12.9%	151	17.9%	80.2%		72.0%	
pT category (7th)											
pT 2	128	8	6.2%	20	15.6%	26	20.3%	78.5%	<0.0001	82.8%	<0.0001
pT 3	2851	141	8.1%	253	14.4%	353	20.2%	79.2%		83.4%	
pT 4	2242	202	9.0%	700	31.2%	836	37.3%	61.3%		68.1%	
pN category (7th)											
pN 0	2070	106	5.1%	252	12.2%	332	16.0%	83.6%	<0.0001	87.9%	<0.0001
pN 1a	533	39	7.3%	121	22.7%	149	28.0%	71.4%		74.3%	
pN 1b	539	43	8.0%	187	34.7%	215	39.1%	57.8%		64.8%	
pN 1c	282	42	14.9%	47	16.7%	87	30.9%	69.9%		75.2%	
pN 2a	374	56	15.0%	171	45.7%	207	55.3%	39.5%		50.1%	
pN 2b	323	65	20.1%	195	40.6%	225	69.7%	25.8%		32.9%	
npN category											
npN 0	2070	106	5.1%	252	12.2%	332	16.0%	83.6%	<0.0001	87.9%	<0.0001
npN 1a	526	32	6.1%	116	22.1%	141	26.8%	72.4%		76.2%	
npN 1b	629	51	8.1%	179	28.5%	207	22.9%	65.6%		69.1%	
npN 2a	495	72	14.5%	198	40.0%	252	50.9%	45.7%		57.9%	
npN 2b	401	90	22.4%	228	56.9%	283	60.6%	26.0%		37.1%	
Tumor deposits (TDs)											
Positive	717	131	18.3%	281	39.2%	389	54.3%	44.6%	<0.0001	57.7%	<0.0001
Negative	3404	220	6.5%	692	20.3%	826	24.3%	74.9%		78.9%	
Venous invasion											
Yes	268	47	17.5%	136	50.7%	157	58.6%	36.8%	<0.0001	45.7%	<0.0001
No	3853	304	7.9%	837	21.7%	1058	27.5%	71.7%		77.1%	
Lymphatic invasion											
Yes	26	8	30.8%	10	38.5%	16	61.5%	29.2%	<0.0001	35.6%	<0.0001
No	4095	343	9.4%	963	23.5%	1199	29.3%	69.8%		75.4%	
Differentiation grade											
Well	452	22	4.9%	54	11.9%	73	16.2%	82.7%	<0.0001	88.2%	<0.0001
Moderately	3213	275	8.6%	725	22.6%	922	28.7%	70.4%		75.8%	
Poorly	456	54	11.8%	194	42.5%	220	48.2%	50.0%		57.8%	
Pathological category											
Papillary or tubular adenocarcinoma	3856	325	8.4%	888	23.0%	1121	29.1%	69.9%	<0.0001	75.9%	<0.0001
Mucinous adenocarcinoma	220	20	9.1%	63	28.6%	68	30.9%	68.2%		70.1%	
Signet ring cell cancer	45	6	13.7%	22	49.9%	26	57.8%	40.8%		41.5%	
Histological type											
Protrude	2733	230	8.4%	561	20.5%	722	26.4%	73.0%	<0.0001	78.5%	<0.0001
Ulcer	1151	98	8.5%	322	28.0%	386	33.5%	64.8%		70.1%	
Infiltrative	237	23	9.7%	90	38.0%	107	45.1%	52.1%		61.2%	
Adjuvant therapy											
Yes	2796	228	8.2%	672	24.0%	825	29.5%	70.1%	0.361	76.7%	0.002
No	1325	123	9.3%	301	22.7%	390	29.4%	68.2%		71.8%	

**Table 4:** Multivariate analysis for 5-year DFS and OS when npN category enrolled

Variables	5-Year DFS			5-Year OS		
	HR	95.0% CI	*P*	HR	95.0% CI	*P*
Age	—	—	—	1.371	(1.181 to 1.592)	<0.0001
Preoperative CEA	0.901	(0.792 to 1.024)	0.111	0.970	(0.837 to 1.123)	0.683
PreoperativeCA199	0.987	(0.844 to 1.154)	0.867	0.843	(0.711 to 0.994)	0.041
pT category	1.461	(1.280 to 1.668)	<0.0001	1.533	(1.312 to 1.791)	<0.0001
pN category	1.367	(1.313 to 1.422)	<0.0001	1.462	(1.398 to 1.529)	<0.0001
TDs	0.591	(0.509 to 0.687)	<0.0001	1.103	(1.039 to 1.200)	0.036
Venous invasion	0.729	(0.594 to 0.895)	0.003	0.816	(0.648to 1.027)	0.083
Lymphatic invasion	0.455	(0.276 to 0.750)	0.002	0.555	(0.323 to 0.954)	0.033
Differentiation grade	1.387	(1.209 to 1.591)	<0.0001	1.425	(1.222 to 1.663)	<0.0001
Pathological category	1.112	(1.006 to 1.229)	0.037	1.160	(1.036 to 1.298)	0.010
Histological type	0.924	(0.774 to 1.103)	0.381	1.131	(0.940 to 1.361)	0.193
Adjuvant Chemotherapy	—	—	—	1.660	(1.413 to 1.950)	<0.0001

**Table 5:** Multivariate analysis for 5-year DFS and OS when pN category enrolled

Variables	5-Year DFS			5-Year OS		
	HR	95.0% CI	*P*	HR	95.0% CI	*P*
Age	—	—	—	1.346	(1.160 to 1.563)	<0.0001
Preoperative CEA	0.901	(0.793 to 1.023)	0.108	0.950	(0.822 to 1.099)	0.493
Preoperative CA199	0.976	(0.837 to 1.138)	0.755	0.839	(0.709 to 0.993)	0.041
pT category	1.448	(1.270 to 1.651)	0.000	1.517	(1.300 to 1.769)	<0.0001
npN category	1.519	(1.444 to 1.598)	<0.0001	1.653	(1.560 to 1.752)	<0.0001
TDs	0.665	(0.570 to 0.775)	<0.0001	1.108	(1.007 to 1.202)	0.032
Venous invasion	0.730	(0.595 to 0.897)	0.003	0.791	(0.629 to 0.995)	0.045
Lymphatic invasion	0.466	(0.283 to 0.769)	0.003	0.534	(0.311 to 0.917)	0.023
Differentiation grade	1.333	(1.163 to 1.529)	<0.0001	1.384	(1.187 to 1.613)	<0.0001
Pathological category	1.094	(0.989 to 1.210)	0.082	1.140	(1.108 to 1.277)	0.023
Histological type	0.936	(0.784 to 1.117)	0.463	1.139	(0.947 to 1.371)	0.168
Adjuvant Chemotherapy	—	—	—	1.747	(1.488 to 2.052)	<0.0001

The pN and npN categories were calculated by Harrell's C statistic to identify which one had superior predictive capacity. The npN category (Harrell's C = 0.716, 95% CI: 0.709 to 0.728) was found to be superior to the pN category (Harrell's C = 0.707, 95% CI: 0.695 to 0.718) for DFS. Also, the npN category was a more accurate predictor than pN category for OS (Harrell's C = 719, 95% CI: 0.700 to 0.736; 712, 95% CI: 0.696 to 0.731, respectively). To identify whether one TD and one pLN had the same weight in patient outcome, we compared the 5-year DFS and OS rates for patients with pure pLNs with patients with pLNs plus TDs. The results are shown in Table 6 and indicate no prognostic heterogeneity meaning that TDs had the same weight as the pLNs (all *P* < 0.05).

**Table 6:** Influence of TDs on 5-year DFS and OS in subgroups of npN category

npN Category	No. of All Patients	All Failure with TDs		All Failure without TDs		5-Years DFS Rate	5-Years OS Rate
		No.	%	No.	%		
npN 1a	526	27	29.00%	114	26.30%	72.1% *vs*.73.6%	76.0% *vs*.76.2%
npN 1b	629	73	34.10%	134	32.30%	65.4% *vs*.65.7%	68.9% *vs*.69.9%
npN 2a	495	107	53.20%	145	49.30%	44.8% *vs*.46.3%	57.3% *vs*.58.5%
npN 2b	401	150	71.80%	133	69.30%	25.4% *vs*.27.6%	36.8% *vs*.37.5%

Note: the 5-year DFS rates of the subgroups with or without TDs in npN1a, 1b, 2a, 2b were similar (all P < 0.05), and the 5-year OS rates of the subgroups had the similar results (all P < 0.05).

## DISCUSSION

Changes in definitions of what should be considered as positive lymph nodes (pLNs) and tumor deposits (TDs) have been causing great confusion and having a large impact on patient prognosis and selection for postoperative chemoradiotherapy. Although tumor deposits are defined as focal aggregates of adenocarcinoma located in the pericolic or perirectal fat discontinuous with the primary tumor and unassociated with a lymph node, it is difficult to distinguish TDs and nodes. Thus, TNM5 proposed the 3-mm rule, which defined a tumor nodule > 3mm in diameter without histological evidence of residual lymph node tissue as a TD [1]. However, this rule was reported as being based on unpublished data, which were not substantiated. In TNM6, the new definition of TDs based on contour replaced the 3-mm rule, and defined TDs to be classified as pLNs when they had the form and smooth contour of lymph nodes, while irregular TDs were classified in the pT category and as venous invasion [3]. Still, this contour rule lacks support from clinical evidences and reproducibility is poor because of the absence of appropriate guidelines [8].

Currently, the TNM7 proposed a novel pN category (pN1c) in stage III in the absence of lymph node (LN) metastasis which states T1 and T2 lesions that lack regional positive lymph node(s) but have tumor deposit(s) be classified in addition as pN1c, though it is not consistent in that in pN1c is also an option for pT3/4a tumors in the CRC staging table [10]. However, this new edition does not propose guidelines on the definition of TDs, which might impact reproducibility because of subjective opinion from pathologists. Although the TNM staging system changed several times with lack of appropriate guidelines, it is still the most important determinant of prognosis in CRC and it is the basis for the patient management guidelines that influence most patient management decisions [5].

The prevalence of TDs ranges from 6% to 64% in CRC and 17% to 55% in colon cancer [13]. The TNM7 abandoned the 3-mm rule and retained the contour rule so that classification of TDs is left to the discretion of the pathologists and pN1c is designated for TDs. However, when we investigated the use of the new definition for TDs, we found that all studies selected TDs depending, in part, on the definition. In other words, TDs selection is still lacking in guidelines. Still, it is difficult to distinguish pLNs and TDs especially when nodes are replaced completely by tumor cells. In fact, the TNM7 gastric cancer staging system has considered TDs as metastatic lymph nodes and the number of TDs was included for pathologic staging [12]. Additionally, TDs were considered as pLNs in Japanese classification of CRC [14]. Song YX et al [5] declared that tumor deposits can be counted as metastatic lymph nodes in TNM staging system for CRC. Based on the above evidence, we attempted to consider all TDs as pLNs and re-designate the pN category.

In the present study, we considered all TDs as pLNs and the npN category was determined by the number of pLNs plus TDs. By using the npN category, we simplified the node category and investigated the feasibility and effects. The 5-year DFS and OS rates of patients with or without TDs were 44.6% *vs*. 74.9% and 57.7% *vs*. 78.9% (*P* < 0.0001, respectively), which indicatapproved that patients with TDs had a worse DFS and OS compared with patients without TDs. This result was similar with to a previous study [8]. By using univariateble and multivariateble analyses, we concluded that TDs could be potentially an independent and adverse prognostic factor for colorectal cancer. Of note, in multivariable analysis, we found that both the npN and pN category were independent predictors for long-term outcome, including DFS and OS in CRC. And then we declared that, however, the npN category was superior to the pN category for DFS (Harrell's C = 0.716, 95% CI: 0.709 to 0.728 *vs*. 0.707, 95% CI: 0.695 to 0.718) and OS (Harrell's C = 719, 95% CI: 0.700 to 0.736 *vs*. 0.712, 95% CI: 0.696 to 0.731). Thus, we proposed that the TDs can be counted as pLNs in TNM staging system and the npN category is feasible and superior to the pN category for predicting the long-term outcomes in CRC.

The origins of TDs remain controversial. Some studies propose that 3 types of TDs can be identified. They define TDs as “vascular invasion type,” “TDs other than the vascular invasion type,” and “tumor deposits, extramural venous and perineural types of invasion” [15, 16]. Recent studies have declared strong correlations between the presence of TDs and vascular invasion [7, 17, 18]. However, in the present study, we did not differentiate between types of TDs and reported that 32.5% (87/268) of the TDs was with venous invasion, which was lower than previous studies. Besides, 46.2% (12/26) of the patients with TDs also had lymphatic invasion. In our study, we differentiated venous invasion from lymphatic invasion by hematoxylin-eosin (H-E) staining, which may reduce the accuracy of recognizing venous and lymphatic invasion. In fact, many other factors such as the histological sectioning, which is only a 2-demensional sample of the 3-demensional structure, could cause us to underestimate the association of TDs with vessels.

Whether or not TDs should be considered pLNs or satellite tumor nodules for the purposes of staging may be difficult to determine. It is thus necessary and reasonable to consider TDs as pLNs. Using the npN category, pathologists and clinicians can simplify the TNM staging system and make suitable suggestions for patient postoperative treatment.

The results from this study are constrained by all the flaws and biases inherent to a nonrandomized trial, although this study included large-scale and multicenter data. Additionally, there are no clear and regular guidelines on how to identify the TDs from lymph nodes, which also may potentially influence the conclusions. The ideal trial design to investigate TDs according to sections of whole specimens would be a prospective clinical trial, which may be helpful to get more reliable data.

In sum, we found that it was feasible to consider TDs as positive lymph nodes in the pN category for evaluating the prognoses of CRC patients, and the npN category was potentially superior to the 7th pN category for predicting the disease-free and overall survival of advanced CRC patients. Whether the npN category has any additional significant practical impact on patients management needs more data to validate.

## PATIENTS AND METHODS

### Ethics considerations

Ethical approval was obtained from the appropriate ethics committees of all participating study sites before the enrolment of patients started. Informed consent was obtained by the investigator at each center from all patients before patient enrollment.

### Patients

A total of 4,121 patients with stage II and III colorectal adenocarcinoma who received an initial radical surgery were identified at seven study centers in China between January 2004 and December 2011, and all relevant data were retrospectively collected including age, gender, serum CEA and CA199 levels, date of surgery, location of the primary tumor (colon and rectal), date and site of recurrence, pathological result, adjuvant chemoradiotherapy and cause of death (CRC related or other cause). We defined colon cancer including tumors locating in cecum to sigmoid colon, and rectal cancer containing tumors locating in rectum or rectosigmoid junction according to the definition from Chok KS et al. [19].

The exclusion criteria were as follows: 1) patients who received neoadjuvant chemoradiotherapy (NCRT, resulting in less nodes detection in specimens); 2) patients with distant metastasis such as liver metastasis found pre- or perioperatively; 3) patients with multiple adenocarcinomas of colon and rectum; 4) patients with synchronous or metachronous tumors; 5) patients who had suffered from colorectal cancer before; 6) patients who died in the immediate postoperative period (within 1 month); 7) patients with positive circumferential resection margins; and 8) patients without complete pathological slides.

### Treatments

All patients initially received R0 resection without preoperative radiotherapy and/or chemotherapy. For rectal cancer patients, the standard total mesorectal excision was performed. After surgery, patients were treated with radiotherapy and/or chemotherapy or not according to body situation and TNM staging system except some patients who rejected adjuvant therapy. Patients with rectal cancer received adjuvant chemoradiotherapy (40-50Gy/2Gy/20-25F and Xeloda basically), while colon cancer patients were treated with Xeloda and 5-Fu regimens basically. 1325 patients did not received adjuvant therapy, including 83.1% (1101/1325) of patients who were in low risk, and other 16.9% (224/1325) who were in high risk (venous invasion, lymphatic invasion, poor differentiation, or advanced stage) but rejected adjuvant therapy (46.4%, 104/224), or were in poor body situation and could not tolerate adjuvant therapy (53.6%, 120/224).

### Pathologic examination

Sections from all resected specimens were examined by local pathologists from seven hospitals. The standardized protocol included determination of the AJCC TNM classification, stage grouping, differentiation degree, histological type, pathological number of examined and involved lymph nodes, and presence or absence of lymphatic or venous invasion. All slides were reviewed for the presence of TDs, defined as either macroscopic or microscopic depositions of carcinoma, without any residual lymph node structures. TDs were assessed using the 3-mm (TNM5) and contour (TNM6) rules [7, 8]. For a regular tumor nodule, we classify it as a positive LN. For an irregular node without any residual tissues of LN we consider it as a TD if the diameter > 3mm measured by a ruler, otherwise, we consider the irregular node as pT3 if the diameter ≤3mm. The reference pathologist tested pathological sections and then recorded the findings in a standardized document.

### Classification methods

All patients were classified depending on TNM7, and then we counted TDs as pLNs in a new pN category. In this study, the pN category which combined with the number of TDs was recorded as npN category [5].

### Follow-up

The follow-up result was collected from all seven hospitals'database. The end point of follow-up was May 2015. The median time of follow-up was 66 months (range: 2-136 months).

### Statistical analysis

Local recurrence and distant metastasis analyses were performed for all eligible patients who received R0 resection without distant metastasis found at time of surgery. All time-to-event end points were measured from date of radical surgery. Disease-free survival (DFS) and overall survival (OS) was calculated from radical resection to finding evidence of local recurrence and/or distant metastasis. Statistical analysis was performed using SPSS software (version 19). Differences were evaluated with the log-rank test. Analyses for local recurrence and distant metastasis were calculated as cumulative incidences. Mutivariate models were performed using the Cox proportional hazards model. All significant variables in the univariate analysis were included in multivariate Cox regression models in a forward-step procedure. The variables were entered in the order according to clinical relevance into the regression models with increasing complexity, and significance was assessed using analysis of variance analysis. The predictive power of the individual models was evaluated using Harrell'C statistic. A model with perfect predictive capacity (sensitivity and specificity of 100%) would have a Harrell'C statistic of 1.00 and the highest Harrell'C statistic was chosen as the best model [20]. A two-sided p value less than 0.05 was considered to be significant.
